# Alcohol use, dementia risk, and sex: a systematic review and assessment of alcohol-attributable dementia cases in Europe

**DOI:** 10.1186/s12877-023-03972-5

**Published:** 2023-04-25

**Authors:** Carolin Kilian, Sinja Klinger, Jürgen Rehm, Jakob Manthey

**Affiliations:** 1 Institute of Clinical Psychology and Psychotherapy, Technische Universität Dresden, Dresden, Germany; 2 Institute for Mental Health Policy Research, Centre for Addiction and Mental Health (CAMH), Toronto, ON Canada; 3 Centre for Interdisciplinary Addiction Research, Department of Psychiatry, University Medical Center Hamburg-Eppendorf, Hamburg, Germany; 4 Program On Substance Abuse, Public Health Agency of Catalonia, Barcelona, Spain; 5 Dalla Lana School of Public Health, University of Toronto, Toronto, ON Canada; 6 Campbell Family Mental Health Research Institute, Centre for Addiction and Mental Health, Toronto, ON Canada; 7 Department of Psychiatry, University of Toronto, Toronto, ON Canada; 8 Department of International Health Projects, Institute for Leadership and Health Management, I.M. Sechenov First Moscow State Medical University, Moscow, Russian Federation; 9 Department of Psychiatry, Medical Faculty, University of Leipzig, Leipzig, Germany

**Keywords:** Dementia, Alcohol use, Alcohol use disorder, Sex, Gender

## Abstract

**Background:**

High-risk alcohol use is an established modifiable risk factor for dementia. However, prior reviews have not addressed sex differences in alcohol-related dementia risk. In this systematic review, we take a sex-specific perspective towards the alcohol-dementia link, taking into account the age of dementia onset.

**Methods:**

We searched electronic databases for original cohort or case–control studies investigating the association between alcohol use and dementia. Two restrictions were considered: First, studies had to report results stratified by sex. Second, given the fact that the age at dementia onset seems to affect the alcohol-dementia link, studies were required to distinguish between early-onset and late-onset dementia (cut-off: 65 years). Additionally, the contribution of alcohol to dementia incidence was quantified for a set of 33 European countries for the year 2019.

**Results:**

We reviewed 3,157 reports, of which 7 publications were finally included and summarised narratively. A lower dementia risk when drinking alcohol infrequent or at moderate levels was found in men (three studies) and women (four studies). High-risk use and alcohol use disorders increased the risk of mild cognitive impairment and dementia, particularly early-onset dementia. Estimating the alcohol-attributable share of incident dementia cases revealed that 3.2% and 7.8% of incident dementia cases were estimated to be attributable to high-risk alcohol use (at least 24 g of pure alcohol per day) in 45-to-64-year-old women and men, respectively.

**Conclusions:**

Research to date has paid little attention to the sex-specific link of alcohol and dementia. In the absence of sex-specific research, the established recommendations on high-risk alcohol use should be employed to communicate the alcohol-attributable dementia risk.

**Supplementary Information:**

The online version contains supplementary material available at 10.1186/s12877-023-03972-5.

## Introduction

As of 2019, an estimated 57.4 million people worldwide were living with dementia, and it is predicted that the number of cases will almost triple in the next three decades [1]. With increasing life expectancy, dementia is becoming one of the most prevalent and serious diseases among the elderly. Several modifiable risk factors have been identified and were estimated to explain about 40% in dementia outcomes [2]. These modifiable risk factors include high-risk alcohol use (i.e., weekly consumption of more than 168 g of pure alcohol).

Various systematic reviews and meta-analyses have been published so far suggesting a link between high levels of alcohol use and dementia (e.g., [3–5]). In a recent systematic review of 28 systematic reviews [3], high levels of alcohol use were found to be a risk factor for dementia as well as for structural and functional changes in the brain. Several pathways were suggested to contribute to elevated risk levels, including the neurotoxicity of the ethanol metabolite acetaldehyde, the detrimental effect of alcohol on cardiovascular disease and consequently vascular forms of dementia, thiamine deficiency, and alcohol-related brain injuries. There is also some evidence that moderate levels of alcohol use might be protective for dementia development, however, plausible biological explanations for such a link, except for a potential beneficial effect of ischemic disease [6], are lacking (see also [5]). The consideration of the apolipoprotein ɛ4 (APOE4) genotype may explain some of the discrepancies observed, yet inconsistent findings exist on the interaction of alcohol use and carriers of the APOE4 allele (e.g., [7–10]).

Another obstacle to understanding the link between alcohol use and dementia is due to a number of methodological limitations frequently observed when using case–control or cohort studies to identify risk factors (see [3, 11, 12]). These limitations concern the underestimation of alcohol use relative to *per capita* consumption [13, 14], inconsistent definitions of alcohol consumption patterns and abstinence [15], regression dilution bias due to measurement error when alcohol use is surveyed only once [12], and missing consideration of confounders, such as APOE4 genotype, education, and other known dementia risk factors such as smoking and physical inactivity [2]. Perhaps most importantly, many studies lack the consideration of sex. However, both alcohol use [16] and the risk of developing a dementia are known to vary by sex [17, 18]. Finally, age also seems to factor into the alcohol-dementia link, as alcohol use disorder has been found to be a risk factor for an early onset of dementia in particular [19]. For populations aged 65 and older, the impact of alcohol might be masked by an increasing dementia and mortality risk driven by other factors.

While there is a solid evidence base suggesting high-risk alcohol use is causally linked to dementia, in particular for early-onset dementia, this relationship is not considered in global comparative risk assessments of the Global Burden of Disease study or of the World Health Organization (for a comparison, see [20]). To the best of our knowledge, the only available estimates were performed as part of an assessment of multiple risk factors with alcohol exposure data drawn from an English survey [2].

Against this backdrop, our systematic review will be the first taking a sex-specific perspective to investigate the association between different levels of alcohol use and dementia risk, accounting for the age of dementia onset. In addition, we will estimate the sex-specific contribution of alcohol to incident dementia cases in selected European countries in 2019.

## Methods

### Systematic literature search and synthesis

A systematic literature search in the databases “Web of Science”, “PsycINFO”, “MEDLINE” and “Embase” was conducted, including search terms specifying the outcome (e.g., dementia or cognitive impairment), the exposure to alcohol, and the study design (e.g., cohort or case–control; see Additional file Table 1). The search took place in 10/2021 and included all publications since 01/01/2000. No restrictions were made with regard to the publication language but search terms were in German and English only. Search strategy and study protocol were preregistered with PROSPERO (Unique Identification Number: CRD42021281614).

Identified reports were screened for their eligibility for inclusion in a two-step procedure. First, a pre-selection was made based on titles and abstracts. Second, full texts were screened for relevant data with predefined criteria determining inclusion or exclusion (see Table 1). To explore the sex-specific risk relationship between alcohol use and dementia, we included only studies that reported their results stratified by sex (or be available for women or men only). In addition, the outcome was to be early- and/or late-onset dementia, with a cut-off age of 65 years for early-/late-onset dementia [21]. For studies that did not distinguish between early-/late-onset dementia or did not report the age of dementia diagnosis in the sample, we assumed that dementia cases were of early onset if the maximum age at the end of follow-up was 69 years (65 + 4 years), while we assumed that dementia cases were of late onset if the minimum age at baseline was 61 years (65-4 years). This 4-year tolerance may have resulted in very few dementia cases being misclassified as early- or late-onset dementia. Studies reporting clinical dementia diagnoses, namely Alzheimer’s disease (International Classification of Disease—10th version [ICD-10] F00 [21]), vascular dementia (ICD-10 F01), or all-cause dementia (including clinical dementia diagnosis not otherwise specified), as well as mild cognitive impairment (MCI; ICD-10 F06.7) were eligible for inclusion. Dementia diagnoses (including MCI) were required to be ascertained by clinical assessment; studies in which cognitive impairment was based on self-report or neurocognitive testing not reflecting a clinical diagnosis were excluded.

**Table 1:** Inclusion and exclusion criteria

Criterion	Inclusion	Exclusion
Outcome	All-cause dementia (clinical dementia diagnosis not otherwise specified) Alzheimer’s disease (ICD-10 F00) Vascular disease (ICD-10 F01) Mild cognitive impairment (MCI, ICD-10 F06.7)	No clinical dementia diagnosis or MCI diagnosis (e.g., subjective cognitive impairment) Other dementia diagnosis (e.g., ICD-10 F02: dementia in other diseases classified elsewhere)
Exposure	Alcohol intake, must include lifetime and/or current abstainers Alcohol abuse (AA) or alcohol use disorder (AUD; ICD-10 F10.1, F10.2)	Alcohol intake in combination with other risk factors (e.g., only combined risk of alcohol and tobacco use)
Design	Cohort study Case–control study Prospective study	Cross-sectional studies Qualitative studies including individual case reports
Sample	Adults aged at least 18 years at baseline/before diagnosis	
Results	Main outcome: risk measure for dementia by alcohol intake levels or AA/AUD diagnosis (e.g., odds ratio, risk ratio, hazard ratio) Risk measure must be reported by sex Risk measure must be reported by age of dementia diagnosis: for early-onset dementia: maximum age at the end of follow-up 69 years, for late-onset dementia: minimum age at baseline 61 years Any information that can be used to calculate relevant risk measures	No relevant information reported Results not stratified or available by sex and/or age of dementia diagnosis (i.e., early- vs. late-onset dementia)
Language	No restrictions, search conducted in English and German only	None [studies written in languages other than German/English would have been translated and included]
Time	Published since January 1^st^, 2000	Publication date before January 1^st^, 2000

*ICD-10* International Classification of Disease—10^th^ version

The screening was performed by three independent reviewers (CK, JM, SK) using the software Covidence [22]. Conflicting decisions were discussed and resolved in group discussions. Inter-rater reliability for the full-text screenings for SK and CK, who screened most of the publications (> 75%), was good with 64.7 – 65.8% (Cohen’s Kappa, [23]).

Key information from the included reports, including the study design, baseline and follow-up assessments, key results, and model adjustments, were extracted and either summarised narratively or by means of random-effects meta-analysis, if a minimum number of five studies with a similar outcome (e.g., all-cause dementia, MCI) was available.

### Risk of bias assessment

Risk of bias was evaluated for each report concerning study population, methods, and presentation of results. For this, the “Newcastle – Ottawa Quality Assessment Scale” (NOQAS) for cohort and case–control studies was used [24] with a score range of 0 to 9 (higher scores indicating higher study quality).

### Estimating the sex-specific contribution of alcohol to incident dementia in Europe

Estimating the sex-specific contribution of alcohol to dementia was a post-hoc decision and not part of the pre-registration. The estimation followed through the standard methodology employed for other comparative risk assessments (see e.g., [20]), which combines sales and survey data in order to obtain estimates of alcohol exposure that are less prone to survey biases [25], such as underreporting [14]. It was aimed to estimate the contribution of alcohol to dementia by sex and age group.

The following data sources were employed: sex-specific *per capita* consumption of pure alcohol (APC) in 2019, corrected for spillage (-10%; [26]), the prevalence of past-year alcohol use, and average drinking levels in 33 European countries from a survey of 54,435 respondents (*Developing and Extending Evidence and Practice from the Standard European Alcohol Survey* [DEEP SEAS] project, for a brief sample description, see Additional file Table 2 [27]), population data from the United Nations Population Prospects [28], and the incidence of Alzheimer’s disease and other dementias (ICD-10: F00-F02.0, F02.8-F03.91, F06.2, G30-G31.1, G31.8-G32.89 [29]) from the Institute for Health Metrics and Evaluation [30]. Alcohol-attributable dementia cases were estimated for all countries with available *per capita* consumption and current survey data [27], which included all 27 member states of the European Union plus Bosnia and Herzegovina, Iceland, Moldova, Norway, Serbia, and the United Kingdom (UK).

In order to obtain sex- and age-specific measures of alcohol exposure, the sex-specific APC was distributed to four age groups (15–34; 35–44; 45–64; 65+) based on the survey data. As the survey was restricted to the population aged 18–64, we had to use the following two assumptions: 1) the age group 15–34 is an accurate representation of 15–17-year-olds; and 2) in the age group 65 + prevalence of use and daily drinking levels are at 75% of the levels reported by age group 45–64 of the same country and sex (proportion aligned with global survey estimates of drinking behaviour: [31]; for decreasing alcohol use with age, see [32]).

The sex- and age-specific alcohol exposure indicators were used to derive estimates of high-risk drinking, i.e., the level of chronic alcohol use assumed to be causally linked to dementia incidence (i.e., 24 g pure alcohol per day/168 g per week according to [2]). Using the classic formula to calculate population attributable fractions (PAF, see Formula 1, [33]), these prevalence estimates of high-risk drinking (${P}_{e}$) were combined with relative risk ($RR$) estimates from [2] (RR: 1.18; 95% CI: 1.06 to 1.31). We resorted to using sex-unspecific pooled risk estimates from a previous meta-analysis because sex-specific pooled estimates were not available as we were unable to meta-analyse the identified studies.

(1) $$PAF =\frac{{P}_{e}(RR-1)}{1+ {P}_{e}(RR-1)}$$

Uncertainty intervals for the PAFs were estimated by accounting for uncertainty in APC and relative risk estimates. With bootstrapping methods, 1,000 samples of APC and relative risk estimates were drawn and combined to calculate the PAF. From the distribution of 1,000 PAFs, the median and values between the 2.5% as well as 97.5% quantile were selected to obtain the point estimate and the corresponding uncertainty interval. When combining the PAFs with incident cases of all-cause dementia to calculate the number of alcohol-attributable cases, the uncertainty of the incident estimates was also considered using the same bootstrapping approach.

All data and the corresponding R code [34] used to quantify the contribution of high-risk alcohol use to dementia (including estimation of uncertainty intervals) have been made publicly available [35].

## Results

**Fig. 1:**
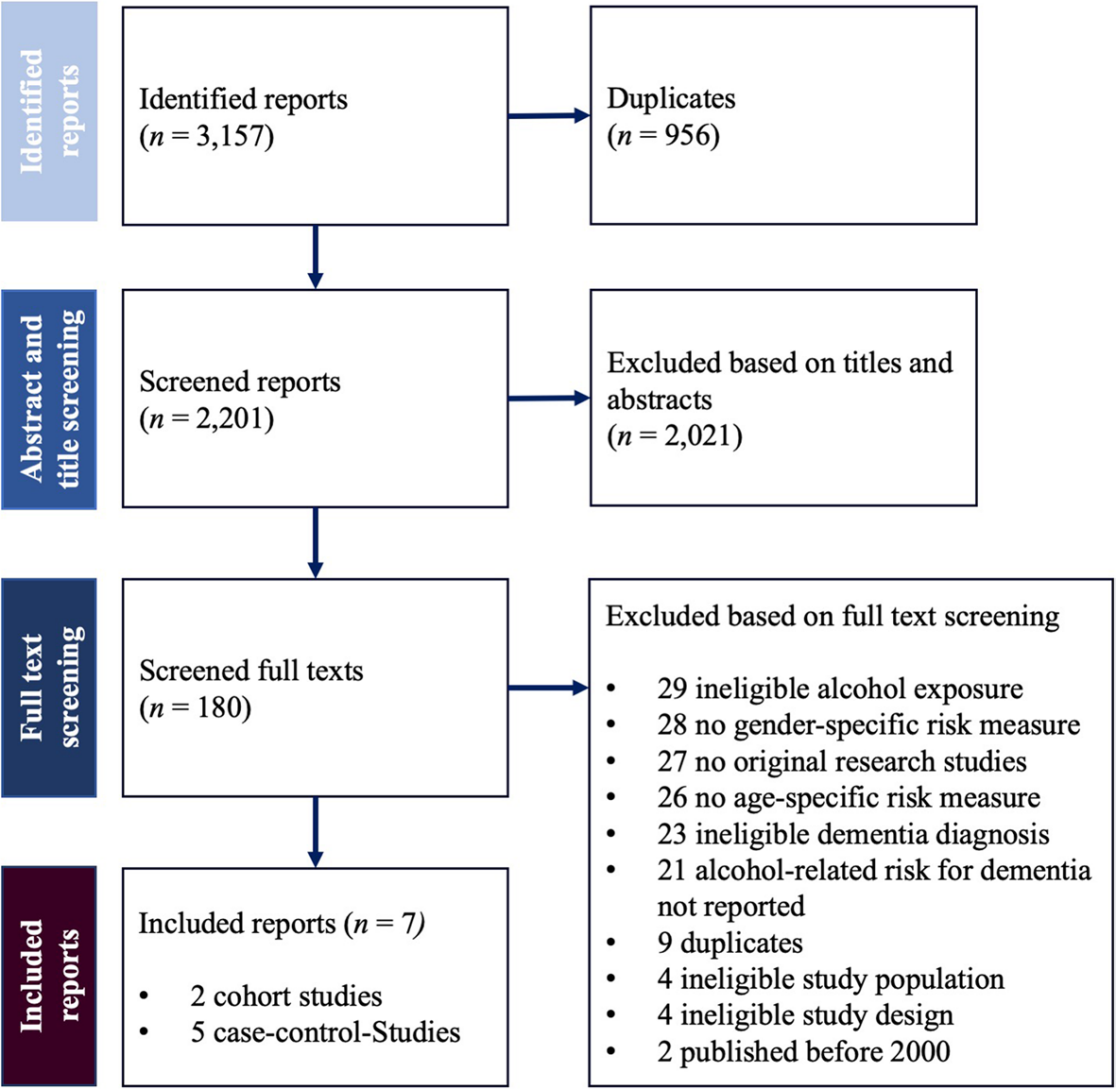
Flowchart of the study selection process

Our systematic literature search yielded 3,157 research reports of which seven fulfilled our inclusion criteria and were therefore included in our systematic review (see Fig. 1). Research reports were most frequently excluded because of their definition of alcohol use (e.g., no consideration of abstinence; *n* = 29), reporting no sex-specific risk measures (*n* = 28), their publication type (e.g., conference abstract; *n* = 27), or a lack of differentiation between early- or late-onset dementia (*n* = 26). An overview of the included reports is given in Table 2 and details on each study’s risk of bias are available in Additional file Table 3 (cohort studies) and 4 (case–control studies). All but one study [19] investigated late-onset dementia.

**Table 2:** Studies included in the systematic review

Publication	Country (region/city)	Study design	Study period	Sample size	Mean age at baseline (standard deviation)	Outcome (number of incident cases)	Classification of exposure	Key findings
Level of alcohol use and the risk of dementia								
Simons et al. 2006 [36]	Australia (Dubbo)	Cohort	Baseline: 1988 – 1989 Follow-up: 1990 – 2004	2,805	60 +	All-cause dementia (not otherwise specified; *n* = 285)	Any or no alcohol use (no reference period)	Any alcohol use (compared to no alcohol use) was associated with decreased dementia risk among women but not among men
Liu et al. 2018 [37]	Japan (Okayama City)	Cohort	Baseline: 2006 – 2007 Follow-up: 2014 (registry data)	53,311	71.4 (7.5)	All-cause dementia (not otherwise specified; *n* = 2,172)	No current use, infrequent or daily use of ≤ 2 drinks (i.e., ≤ 40 g of pure alcohol) per day, and infrequent or daily use of > 2 drinks per day (i.e., > 40 g of pure alcohol)	Drinking ≤ 2 drinks per day or infrequent drinking was associated with reduced dementia risk (compared to no current use) among both men and women
Mukamal et al. 2003 [38]	USA	Case–control	Baseline: 1992 – 1994 Follow-up: 1997 – 1999	746	Cases: 77.8 (5.5), controls: 77.5 (5.2)	All-cause dementia (not otherwise specified; *n* = 151), Alzheimer’s disease (*n* = 130), vascular dementia (*n* = 36)	Weekly alcohol use: abstinence five years before baseline, former alcohol use, cessation of use during follow-up, current weekly drinking of < 1, 1–6, 7–13, and ≥ 14 drinks (one drink contains 14 g of pure alcohol [39])	Moderate levels of weekly alcohol use were associated with reduced dementia risk (compared to abstinence) in men and women; drinking levels linked to dementia risk varied between men and women
Larsson & Wolk 2018 [40]	Sweden	Cohort	Baseline: 1997 Follow-up: 1998 –2014 (registry data)	28,775	71.6 (4.5)	Alzheimer’s disease (*n* = 3,755)	Weekly alcohol use: lifetime abstinence, former alcohol use, current weekly drinking of < 1, 1–6, 7–14, 14–21, and > 21 drinks (one drink contains 12 g of pure alcohol)	No significant association between different levels of weekly alcohol use and dementia risk (compared to lifetime abstinence) among both men and women
Takahashi et al. 2011 [41]	USA (Olmsted County)	Case–control	1994 – 2002 (registry data)	410	Cases: 82.1 (7.7), controls: 81.6 (7.9)	Vascular dementia (*n* = 205)	Current, previous, or ever alcohol use (categories not independent of each other)	Reduced dementia risk among men and women with current alcohol use (compared to no current alcohol use)
Level of alcohol use and the risk of MCI								
Anttila et al. 2004 [42]	Finland (Kuopio and Joensuu)	Cohort	Baseline: 1972–1977 Follow-up: 1998	1,018	48.3 (4.8)	MCI (*n* = 61)	No, infrequent (less than once a month), or frequent (several times a month) alcohol use	Increased MCI risk for frequent (compared to infrequent) alcohol use in men but not in women, risk was not increased for infrequent (compared to no) alcohol use in both men and women
Alcohol use disorder and the risk of dementia								
Schwarzinger et al. 2018 [19]	France	Cohort	Baseline: 2008–2010 Follow-up: 2011–2013	19,769,440	Men: 55 (40–68), women: 49 (31–67)*	All-cause dementia (*n* = 503,516), vascular dementia (*n* = 173,813), MCI (*n* = 178,951), early-onset dementia (*n* = 27,566)	Alcohol use disorder (ICD-10: F10.1-F10.9, Z50.2) or chronic disease attributable to alcohol use disorder (e.g., K70)	Presence (compared to absence) of alcohol use disorder was associated with higher risks of MCI and all-cause dementia in men and women; higher risks for early dementia onset (age: < 65)

^*^Median age and interquartile range provided. *MCI* Mild cognitive impairment, *ICD-10* International Classification of Disease-10th Revision [21]. *USA* United States of America

### Level of alcohol use and the risk of dementia

In a first cohort study, 2,805 adults aged 60 years and older from Dubbo (Australia) were followed-up for 16 years [36]. Baseline assessment took place in 1988, including a questionnaire on alcohol use (any vs. no use, reference period not reported), and none of the participants had any cognitive impairment at this point of time. At the end of the follow-up, 285 dementia cases were recorded. As adults were 60 + at baseline and followed-up over several years, we have specified these dementia cases as late-onset dementia, although the age of dementia onset was not reported in this study. Compared to women reporting no alcohol use at baseline, those indicating any alcohol use had a 33% decreased risk for dementia after 16 years (hazard ratio [HR] = 0.67, 95% confidence intervals [CI]: 0.48 – 0.93), adjusting for marital status, education, a history of stroke, and activities of daily living. In men, however, the link between alcohol use and dementia development was not significant (0.64, 95% CI: 0.41 – 1.01). While the overall risk of bias was low (NOQAS score: 7/9 points), alcohol use was recorded in self-report and lifetime abstainers were mixed with former alcohol users.

A Japanese cohort of 53,311 older adults (mean age: 71.4 years) were followed-up over a seven-year period [37]. Age-standardised prevalence of dementia at the end of the follow-up was 13.4%. Age at dementia onset was again not reported in this study, but given the high mean age at baseline, dementia cases were considered to involve largely late-onset dementia. Among men, occasionally or daily drinking of ≤ 2 drinks was associated with a reduced dementia risk (occasionally: HR = 0.88, 95% CI: 0.81 – 0.96, daily: HR = 0.79, 95% CI: 0.73 – 0.85) compared to current abstainers. A similarly protective effect of consuming ≤ 2 drinks per day emerged for women (occasionally: HR = 0.84, 95% CI: 0.79 – 0.90, daily: HR = 0.87, 95% KI: 0.78 – 0.97). Compared to current abstainers, drinking more than two drinks per day was not associated with an increased dementia risk among men (occasionally: HR = 0.91, 95% CI: 0.71 – 1.16, daily: HR = 0:89, 95% CI: 0.81 – 1.00) and women (occasionally: HR = 1.09, 95% CI: 0.72 – 1.67, daily: HR = 1.16, 95% CI: 0.84 – 1.81). Models were adjusted for age, as well as for different health indicators and lifestyle risk factors. Study quality was moderate (NOQAS score: 6/9 points), however, only current alcohol use was captured and no baseline cognitive assessment was carried out. To account for the latter, the authors conducted a sensitivity analysis excluding participants developing dementia in the first two years of follow-up, yielding similar study findings.

A US case–control study compared 373 adults aged 65 years or older having a dementia diagnosis (i.e., all-cause dementia, Alzheimer's disease, and vascular dementia) with 373 healthy controls of the same age, mortality, and presence in follow-up profile [38]. Cases and controls were taken from a cohort (Cardiovascular Health Study, *n* = 5,888 adults), with a median follow-up time of about six years. Weekly alcohol use was recorded using self-report at baseline, asking about any use five years before the assessment, as well as in follow-ups, and were averaged across measurement points. Reporting a weekly intake of 1–6 alcoholic drinks was associated with a reduced all-cause dementia risk among men (odds ratio [OR] = 0.36, 95% CI: 0.17 – 0.77), while among women, the risk was reduced when consuming either less than one drink per week (OR = 0.52, 95% CI: 0.30 – 0.90) or 7–13 drinks per week (OR = 0.23, 95% CI: 0.09 – 0.61), compared to abstainers of the same sex. A similar protective effect of consuming 1–6 drinks per week in men and less than one drink per week in women, compared to abstaining from drinking alcohol, was observed for Alzheimer’s diseases, but not for vascular dementia. No differences in dementia risk were found between those who reported abstaining from alcohol five years before baseline and those who either consumed alcohol before but not at baseline (i.e., former drinkers), stopped drinking during follow-up (i.e., quitters), consumed ≥ 14 drinks per week, or consumed alcohol at any other level. Models were adjusted for age, APOE4 genotype, and different health indicators. In additional non-sex-stratified analyses, this study also assessed the risk for all-cause dementia from drinking alcohol stratified by APOE4 genotype finding that the aforementioned protective effect of consuming less than one and 1–6 drinks per week was only evident in those who were *no* carriers of the APOE4 allele. Study quality was rated as good (NOQAS score: 8/9 points).

Alzheimer’s diseases were also investigated in a Swedish cohort of 28,755 adults aged 65 to 83 years, which was followed-up over a mean time of 12.6 years [40]. Self-reported alcohol use was captured in the baseline assessment in 1997 and the following drinking patterns were considered: lifetime abstinence, former alcohol use, current weekly consumption of ≤ 1 drink, 1–6 drinks, 7–14 drinks, 14–21 drinks, and more than 21 drinks. Alzheimer’s disease diagnoses were obtained from registry data (1998 – 2014) and linked to the questionnaire data. In total, 3,755 diagnoses were registered in the study population. No significant association between different levels of alcohol use (reference group: lifetime abstainers) and the risk of Alzheimer's disease was established for both men and women, adjusting for age, education, as well as for different health indicators and lifestyle risk factors. Risk of bias was low (NOQAS score: 7/9 points).

In another US case–control study, the link between alcohol use and vascular dementia was explored, comparing 205 patients with 205 healthy controls of the same sex, age (mean: 81.9 years), and access time to the medical system (based on the person’s hospital registration number) [41]. Three drinking patterns were distinguished based on self-report: alcohol use at any time in life, former alcohol use, and current alcohol use. Importantly, drinking patterns were not independent of each other, as current and former alcohol users were considered to have consumed alcohol at any time in life. Sex-stratified analysis revealed a protective effect of current alcohol use in women (OR = 0.45, 95% CI: 0.25 – 0.84) and men (OR = 0.52, 95% CI: 0.28 – 0.96), compared to those not having consumed alcohol at the time of the assessment. No significant link between the other drinking patterns and vascular dementia risk were found. Study quality was moderate (NOQAS score: 6/9 points), but the interpretation of results is limited as confounders were not controlled for.

### Level of alcohol use and the risk of MCI

We identified only one research report exploring the link between alcohol use and MCI in a cohort of 1,464 adults from Finland [42]. Self-reported alcohol use (abstainers, less than monthly, or several times per month, reference period not reported) was recorded at baseline, while the cognitive assessment took place on average 23 years later. In the overall sample, 61 cases of MCI and 48 of dementia were identified. In men, the odds of MCI were increased among those reporting frequent alcohol use compared to those indicating to drink less than monthly (OR = 5.08, *p* = 0.020), while the odds were not significantly different between abstainers and infrequent users (OR = 3.84, *p* = 0.120). In women, MCI risk did not significantly differ across drinking patterns (several times per months vs. infrequent use: OR = 1.63, *p* = 0.430; abstaining vs. infrequent use: OR = 1.41, *p* = 0.440). An interaction of alcohol use and sex for dementia risk were not reported. Models were adjusted for age, education, lifestyle and vascular factors. This study also explored the combined effect of alcohol use and APOE4 genotype on dementia risk in the overall sample. Participants who were carriers of the APOE4 allele and consumed alcohol infrequently or frequently had a significantly increased risk of developing dementia compared to non-carriers who abstain from drinking alcohol. However, no such interaction was identified for MCI. Risk of bias was evaluated as low (NOQAS score: 8/9 points).

### AUD and the risk of dementia

Finally, one study looked at the link between AUD and dementia [19]. Data on almost 20 million adults aged 20 years and older were obtained from the French National Hospital Discharge database, including about half a million incident cases of dementia. Among the 512.5 thousand men and 170 thousand women with AUD, 30 and 12.7 thousand were diagnosed with dementia, respectively. Dementia risk was elevated among men (HR = 2.73, 95% CI: 2.68 – 2.78) and women (HR = 2.77, 95% CI: 2.70 – 2.83) aged 65 years and older with AUD (compared to those without an AUD), adjusting for a range of confounders, including vascular risk factor and cardiovascular diseases. Importantly, this study accounted for the patient’s age and found that the presence of AUD is particularly relevant for early-onset dementia, as the hazard ratios were much higher at younger ages (< 65 years), while they decreased with older age (e.g., men aged 85 years and older: HR = 1.78, 95% CI: 1.70 – 1.87; women: 1.79, 95% CI: 1.70 – 1.90). In the overall sample including those younger than 65 years, an increased risk for MCI was found among men (HR = 2.43, 95% CI: 2.37 – 2.49) as well as among women (HR = 2.29, 95% CI: 2.22 – 2.36). Study quality was good (NOQAS: 8/9 points).

### Incident alcohol-attributable dementia cases in Europe

The identified studies do not suggest that heavy alcohol use is associated with an increased risk for dementia at older ages (65 years or older) [37, 40]. Furthermore, one study suggested that the elevated risk for AUD declines with age [19]. To be conservative and to avoid overestimates, we limited the estimation of alcohol-attributable dementia cases to adults aged 45–64 years, in line with previous work [2].

**Fig. 2:**
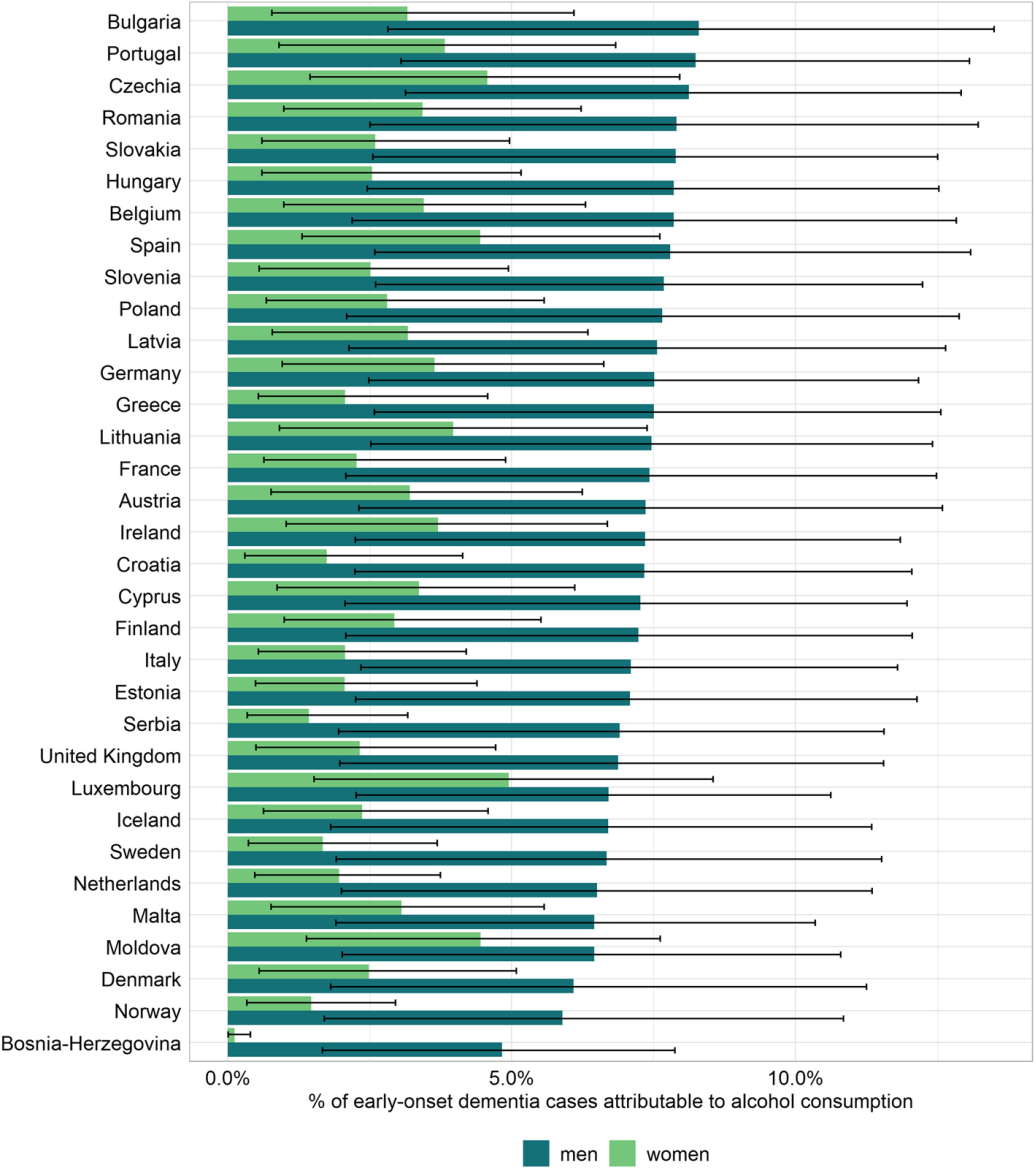
The proportion of dementia cases in 2019 occurring among 45–64-year-olds that are attributable to high-risk alcohol use

Of 67,726 all-cause dementia cases among 45–64-year-olds, high-risk alcohol use was estimated to be linked to 3,536 incident dementia cases in the 33 European countries in 2019 (women: 1,196, 95% CI: 820 to 1,701; men: 2,340, 95% CI: 1,679 to 3,164). The contribution of alcohol varied across countries, with shares ranging mostly between 1 and 5% among women (mean share weighted for the number of incident cases: 3.2%) and between 5 and 8% among men (mean share weighted for the number of incident cases: 7.8%; see Fig. 2).

## Discussion

Our systematic review is the first sex-stratified assessment of studies exploring the link between alcohol use and dementia. The findings from seven research reports revealed a fragmented picture, likely in part due to known methodological issues (see [3] and limitations below). In summary, three reports found a protective effect of infrequent or moderate levels of alcohol use in men, while this was the case for women in four studies. In contrast, high levels of alcohol use increased the risk of MCI and dementia in men and women, and the presence of AUD was linked to a higher dementia risk in general and to early-onset dementia in particular. In a selection of 33 European countries, we estimated that 2,340 incident dementia cases in men and 1,196 cases in women among the 45–64-year-olds were attributable to high-risk alcohol use in 2019.

The findings of our review show that sex-specific analyses are a rarity in the field of alcohol and dementia research. This phenomenon is not specific to dementia research but has been observed in other contexts, such as in other dose–response meta-analyses for alcohol [43] or in cohort studies on substance use disorders [44]. Conducting sex-specific analyses is essential not only to uncover differences in disease risk between men and women, but also to unmask sex-specific disease mechanisms. At this stage, it is not possible to ascertain whether men differ from women in the association between alcohol use and dementia. However, a sex difference is highly likely given the considerable differences in the alcohol metabolism between both sexes, resulting in higher harm for many health outcomes for the same level of alcohol in women [45, 46]. In addition, there are well-established sex and gender differences in dementia risk factors which could be impacted by alcohol use (e.g., hypertension, increased blood sugar) [18, 47]. We therefore encourage future researchers to perform sex-specific analyses when examining the dementia risk relative to alcohol use.

Under consideration of the evidence from previous reviews and meta-analyses [3–5], moderate levels of alcohol use may have a protective effect on dementia development, while high levels of alcohol use are likely to increase the risk for dementia substantially. Given that the excessive and chronic use of alcohol—or more precisely of its key component ethanol—can cause structural and functional brain damages through various mechanisms (e.g., the direct neurotoxic effect of its metabolite acetaldehyde), its detrimental effect on cognitive functioning is highly biologically plausible [5, 48]. The fragmented picture of the sex-specific evidence seen in our review could be explained by at least four limitations: First, alcohol use was always recorded using self-report, except when AUD was used as exposure. Underestimation of alcohol use, as observed in cohort [13] or survey studies [14], might not be an issue for risk assessments when all individuals would underestimate their alcohol use in a similar fashion. However, previous research has shown that alcohol users with high drinking levels tend to underestimate their alcohol use to a greater extent [14]. Second, all but one study investigated late-onset dementia. As shown in the study by Schwarzinger et al. [19], however, the attributable fraction of dementia caused by alcohol decreases with age. Future studies should define middle-aged adults (45–64 years) as the target population to examine early-onset dementia in particular. Third, only two studies accounted for individual’s APOE4 genotype, which was identified as genetic risk factor for dementia in general [49] and may also have a moderating effect on the alcohol-dementia link [7, 38, 42]. Fourth, some studies failed to distinguish between lifetime abstainers and former drinkers, which is a known limitation of studies researching the risk relationship between alcohol use and health outcomes. This is problematic as many current abstainers are not using alcohol anymore because of former alcohol use problems and consequently this group has a higher mortality risk than low or moderate drinkers [50].

To the best of our knowledge, the PAF of high-risk alcohol use to dementia has only been estimated based on survey data combined with meta-analysed risk relations from three cohort studies, as reported in the 2020 dementia report from the Lancet commission [2]. As we were unable to meta-analyse sex-specific risk estimates given the low number of studies included in this review, we referred to the sex-unspecific risk estimate from the previous meta-analysis [2]. Consequently, our approach assumed the same risk for a given level of alcohol intake for both men and women. This assumption is based on the lack of more granular data and in light of well-established biological and sex differences with regards to alcohol intake, this assumption should be tested in further cohort studies.

Considering these limitations, our results can be considered the first quantification of the contribution of high-risk alcohol use to dementia in Europe. For these estimates, we assumed that high-risk alcohol use is causally related to dementia onset. A causal link is biologically plausible and has been demonstrated for various forms of dementia, and several prospective studies have shown temporality [3].

Our method corrects for underreporting of alcohol consumption in surveys (e.g., [13, 14]), which explains the higher estimate for the UK (2.1% in [2]; women: 3.2% and men: 7.8% in this study). The limitation of the available data is that the same risk for all people drinking at or above high-risk levels was used. As considerably higher risks for dementia were reported for people with AUD [19], our estimates should be interpreted cautiously as they are probably underestimates.

While we only estimated the contribution of alcohol for early-onset dementia, the employed risk estimates of the meta-analysis [2] included dementia diagnoses of older ages (mean age at follow-up or dementia onset is between 75 and 83 years in the three underlying studies: [51–53]). Importantly, in all three studies, alcohol consumption was measured during midlife (at around age 50). As the contribution of alcohol to dementia decreases with increased age [19, 52], these risk estimates may underestimate the contribution of alcohol for dementia that occurs before the age 65. Also, using a sex-unspecific risk estimate may also underestimate the effect of alcohol for women, which are known to experience higher levels of harm at the same level of alcohol consumption, when compared to men (for example, sex-specific risk functions for liver cirrhosis: [54]). Overall, we believe that the actual contribution of alcohol to early-onset dementia may be higher than estimated in this study, which followed a conservative approach.

One aspect that we were not able to account for in the assessment of alcohol-attributable dementia cases is the possibly mediating role of APOE4. Despite these limitations, we believe that the presented results are robust estimates for the contribution of alcohol to early onset of all-cause dementia.

The strongest conclusion of our systematic review is that further sex-specific research is needed on the link between alcohol use and dementia. This research should concentrate on early-onset dementia and distinguish between different types of dementia and between different forms of high-risk drinking including AUD. To correct for potential biases of subjective reporting, biomarkers may serve to measure longer-term exposure to high-risk alcohol use [55]. Future research should also assess the APOE4 genotype more systematically, given that two of the identified studies support its potential moderating role in the alcohol-dementia relationship, however, without considering sex [38, 42]. In the meantime, that is, before a more complex picture may emerge, the guidance of the Lancet Commission to limit alcohol use to less than 168 g of pure alcohol per week [2] should be applied in prevention for both sexes, and in treatment for dementia, alcohol use and history of alcohol use and AUD should be regularly assessed, together with other risk factors.

## Supplementary Information

**Additional file 1.**

## Data Availability

All data processed for estimating the contribution of high-risk alcohol use to dementia incidence, including the corresponding R code, has been made accessible in a public repository: https://doi.org/10.6084/m9.figshare.19690906.v1.

## Abbreviations

APC: Alcohol per capita consumption
APOE4: Apolipoprotein ɛ4
AUD: Alcohol use disorder
HR: Hazard ratio
ICD-10: International Classification of Disease 10^th^ version
MCI: Mild cognitive impairment
NOQAS: Newcastle – Ottawa Quality Assessment Scale
OR: Odds ratio
PAF: Population attributable fraction
RR: Relative risk
UK: United Kingdom
