# Supplementary material for: Alcohol use, dementia risk, and sex: a systematic review and assessment of alcohol-attributable dementia cases in Europe
Source: BMC Geriatr. 2023 Apr 25;23:246. doi: 10.1186/s12877-023-03972-5 (PMC10127029; doi:10.1186/s12877-023-03972-5)
Supplement: Supplementary file 1 — Additional file 1. [file 12877_2023_3972_MOESM1_ESM.docx]

# Additional file

**Title:** Alcohol use, dementia risk, and gender: a systematic review and assessment of alcohol-attributable dementia cases in Europe

**Authors:** Carolin Kilian, Sinja Klinger, Jürgen Rehm, Jakob Manthey

[Additional Table 1. Search terms for the systematic literature search in English and German language. 2](#_Toc121466641)

[Additional Table 2. Brief sample description of the DEEP SEAS survey. 3](#_Toc121466642)

[Additional Table 3. Risk of bias assessments for cohort studies. 5](#_Toc121466643)

[Additional Table 4. Risk of bias assessments for case-control studies. 5](#_Toc121466644)

Additional Table 1. Search terms for the systematic literature search in English and German language.

| Web of Science (English) | TOPIC: (dementia OR alzheimer OR cognitive impairment OR MCI)  AND TOPIC: (alcohol* OR ethanol OR drink* OR drunk*)  AND TOPIC: (cohort OR observation* OR case-control OR case control OR prospective)  Limit 1 tp yr = “2000 – current” |
| --- | --- |
| OVID SP (PsycINFO, MEDLINE, Embase; English) | (dementia OR alzheimer OR cognitive impairment OR MCI).TI,AB.  AND (alcohol$ OR ethanol OR drink$ OR drunk$).TI,AB.  AND (cohort OR observation$ OR case-control OR case control OR prospective).TI,AB.  Limit 1 tp yr = “2000 – current” |
| Search terms in German language | THEMA: (Demenz* OR Alzheimer* OR kognitive Beeinträchtigung OR LKB)  UND THEMA: (Alkohol* OR Ethanol OR trink*)  UND THEMA: (Kohort* OR Beobachtung* OR Fall-Kontrol* OR prospektiv*)  Einschränkung: “2000 – heute” |

Additional Table 2. Brief sample description of the DEEP SEAS survey.

| **Country** | **Mode of administration** | **Sample size** | **Proportion of women (%)** | **Proportion of 18-34-year-olds (%)** | **Proportion of 35-44-year-olds (%)** | **Proportion of 45-64-year-olds (%)** |
| --- | --- | --- | --- | --- | --- | --- |
| Austria | CAWI | 3089 | 51.1 (49.3, 52.9) | 33.9 (32.2, 35.6) | 21 (19.6, 22.4) | 45.1 (43.3, 46.8) |
| Belgium | CAWI | 1501 | 51.1 (48.6, 53.6) | 35 (32.6, 37.4) | 21 (19, 23.1) | 44 (41.4, 46.5) |
| Bosnia and Herzegovina | CATI | 1500 | 52 (49.5, 54.5) | 35 (32.6, 37.4) | 32 (29.6, 34.4) | 33 (30.6, 35.4) |
| Bulgaria | CAWI | 3003 | 51 (49.2, 52.8) | 31 (29.4, 32.7) | 24 (22.4, 25.5) | 45 (43.2, 46.8) |
| Croatia | CAWI | 1500 | 52.1 (49.5, 54.6) | 33 (30.7, 35.4) | 22 (19.9, 24.1) | 45 (42.5, 47.5) |
| Cyprus | CATI | 1500 | 51 (48.5, 53.5) | 41 (38.5, 43.5) | 22 (19.9, 24.1) | 37 (34.6, 39.4) |
| Czechia | CAWI | 1500 | 51 (48.5, 53.6) | 32 (29.6, 34.3) | 26 (23.8, 28.2) | 42 (39.5, 44.5) |
| Denmark | CAWI | 1574 | 50.2 (47.7, 52.7) | 35.8 (33.4, 38.2) | 20.1 (18.1, 22.1) | 44.1 (41.7, 46.6) |
| Estonia | CAWI | 2150 | 53.2 (51, 55.3) | 33.8 (31.8, 35.9) | 23.1 (21.3, 24.9) | 43.1 (41, 45.2) |
| Finland | CAWI | 1494 | 51.3 (48.8, 53.8) | 35.2 (32.7, 37.6) | 21.3 (19.2, 23.3) | 43.6 (41, 46.1) |
| France | CAWI | 1708 | 52 (49.6, 54.4) | 34 (31.8, 36.2) | 21 (19.1, 22.9) | 45 (42.6, 47.4) |
| Germany | CAWI | 1508 | 51.1 (48.6, 53.6) | 32.6 (30.3, 35) | 19.8 (17.7, 21.8) | 47.6 (45.1, 50.1) |
| Greece | CAWI | 1521 | 51 (48.5, 53.5) | 30 (27.7, 32.3) | 24 (21.8, 26.1) | 46 (43.5, 48.5) |
| Hungary | CAWI | 2005 | 52.1 (49.9, 54.3) | 32 (29.9, 34) | 25 (23.1, 26.9) | 43 (40.9, 45.2) |
| Iceland | CATI | 1500 | 49 (46.5, 51.5) | 40 (37.5, 42.5) | 22 (19.9, 24.1) | 38 (35.5, 40.5) |
| Ireland | CAWI | 1497 | 50.1 (47.6, 52.7) | 34.6 (32.2, 37) | 25.7 (23.5, 28) | 39.6 (37.2, 42.1) |
| Italy | CAWI | 1503 | 51 (48.5, 53.6) | 29 (26.7, 31.2) | 22 (19.9, 24.1) | 49 (46.5, 51.6) |
| Latvia | CAWI | 1503 | 54 (51.5, 56.6) | 33 (30.6, 35.4) | 22 (19.9, 24.1) | 45 (42.4, 47.5) |
| Lithuania | CAWI | 1517 | 54 (51.5, 56.5) | 33.7 (31.3, 36) | 19.8 (17.8, 21.8) | 46.5 (44, 49.1) |
| Luxembourg | CAWI, CATI | 1506 | 50 (47.5, 52.5) | 36 (33.6, 38.4) | 23 (20.9, 25.1) | 41 (38.5, 43.5) |
| Malta | CATI | 1500 | 49 (46.5, 51.5) | 39.4 (36.9, 41.9) | 23.2 (21.1, 25.4) | 37.4 (34.9, 39.8) |
| Moldova | CATI | 1500 | 53 (50.5, 55.5) | 42 (39.5, 44.5) | 28 (25.7, 30.3) | 30 (27.7, 32.3) |
| Netherlands | CAWI | 1504 | 50 (47.5, 52.5) | 35 (32.6, 37.4) | 19 (17, 21) | 46 (43.5, 48.5) |
| Norway | CAWI | 1496 | 50.2 (47.6, 52.7) | 36.8 (34.4, 39.3) | 21.1 (19, 23.1) | 42.1 (39.6, 44.6) |
| Poland | CAWI | 1560 | 52 (49.5, 54.5) | 34 (31.6, 36.4) | 25 (22.8, 27.2) | 41 (38.6, 43.4) |
| Portugal | CAWI | 1502 | 53 (50.5, 55.5) | 30 (27.7, 32.3) | 24 (21.8, 26.2) | 46 (43.5, 48.5) |
| Romania | CAWI | 1503 | 51 (48.5, 53.6) | 32 (29.6, 34.3) | 24 (21.9, 26.2) | 44 (41.5, 46.5) |
| Serbia | CAWI | 1501 | 51 (48.5, 53.6) | 32.6 (30.2, 35) | 22.8 (20.7, 24.9) | 44.6 (42.1, 47.1) |
| Slovakia | CAWI | 1504 | 51 (48.5, 53.6) | 34 (31.6, 36.4) | 25 (22.8, 27.2) | 41 (38.5, 43.5) |
| Slovenia | CAWI | 1502 | 50 (47.5, 52.6) | 30 (27.6, 32.3) | 24 (21.9, 26.2) | 46 (43.5, 48.6) |
| Spain | CAWI | 1651 | 51.1 (48.7, 53.5) | 28.9 (26.7, 31.1) | 25 (22.9, 27.1) | 46.1 (43.7, 48.5) |
| Sweden | CAWI | 1627 | 50 (47.6, 52.5) | 37 (34.6, 39.3) | 21 (19, 23) | 42 (39.6, 44.4) |
| United Kingdom | CAWI | 1506 | 51 (48.5, 53.5) | 36 (33.6, 38.4) | 21 (18.9, 23.1) | 43 (40.5, 45.5) |

95% confidence intervals in brackets. CATI: computer-assisted telephone interviews. CAWI: computer-assisted web interviews. DEEP SEAS: Developing and Extending Evidence and Practice from the Standard European Alcohol Survey. National samples drawn from existing Kantar panels using three independent quotas (sex, age group, subnational region). Data collection took place between January and March 2021. Data was weighted for the country-specific population distribution by sex, age group, and subnational region.

Additional Table 3. Risk of bias assessments for cohort studies.

| **Publication** | **(1) Selection** | | | | **(2) Comparability** | **(3) Outcome** | | | **Total score (max. 9)** |
| --- | --- | --- | --- | --- | --- | --- | --- | --- | --- |
|  | **Representa-tiveness of cohort** | **Selection of non-exposed cohort** | **Ascertainment of exposure** | **Outcome was not present at start of study** | **Comparability of cohorts: adjustment for covariates** | **Assessment of outcome** | **Follow-up long enough** | **Adequacy of follow-up** |  |
| Antilla et al. 2004 | 1 | n. a. | 1 | 1 | 2 | 1 | 1 | 1 | 8 |
| Larson et al. 2018 | 1 | n. a. | 0 | 1 | 2 | 1 | 1 | 1 | 7 |
| Liu et al. 2018 | 1 | n. a. | 0 | 1 | 2 | 1 | 1 | 1 | 7 |
| Schwarzinger et al. 2018 | 1 | n. a. | 1 | 1 | 2 | 1 | 1 | 1 | 8 |
| Simons et al. 2006 | 1 | n. a. | 0 | 1 | 2 | 1 | 1 | 1 | 7 |

Assessment based on the Newcastle – Ottawa Quality Assessment Scale for cohort studies. n. a. = not applicable.

Additional Table 4. Risk of bias assessments for case-control studies.

| **Publication** | **(1) Selection** | | | | **(2) Comparability** | **(3) Outcome** | | | **Total score (max. 9)** |
| --- | --- | --- | --- | --- | --- | --- | --- | --- | --- |
|  | **Case Selection** | **Representa-tiveness of cases** | **Selection of controls** | **Definition of controls** | **Cases and controls on the basis of design or analysis** | **Ascertain-ment of exposure** | **Method of ascertain-ment** | **Non response rate** |  |
| Mukamal et al. 2003 | 1 | 1 | 1 | 1 | 2 | 1 | 0 | 1 | 8 |
| Takashi et al. 2011 | 1 | 1 | 0 | 1 | 2 | 0 | 1 | 0 | 6 |

Assessment based on the Newcastle – Ottawa Quality Assessment Scale for case-control studies.
